# An Integrated Autophagy-Related Long Noncoding RNA Signature as a Prognostic Biomarker for Human Endometrial Cancer: A Bioinformatics-Based Approach

**DOI:** 10.1155/2020/5717498

**Published:** 2020-12-12

**Authors:** Ziwei Wang, Jun Zhang, Yan Liu, Rong Zhao, Xing Zhou, Hongbo Wang

**Affiliations:** Department of Obstetrics and Gynecology, Union Hospital, Tongji Medical College, Huazhong University of Science and Technology, Wuhan, Hubei 430022, China

## Abstract

Endometrial cancer is one of the most common malignant tumors, lowering the quality of life among women worldwide. Autophagy plays dual roles in these malignancies. To search for prognostic markers for endometrial cancer, we mined The Cancer Genome Atlas and the Human Autophagy Database for information on endometrial cancer and autophagy-related genes and identified five autophagy-related long noncoding RNAs (lncRNAs) (LINC01871, SCARNA9, SOS1-IT1, AL161618.1, and FIRRE). Based on these autophagy-related lncRNAs, samples were divided into high-risk and low-risk groups. Survival analysis showed that the survival rate of the high-risk group was significantly lower than that of the low-risk group. Univariate and multivariate independent prognostic analyses showed that patients' age, pathological grade, and FIGO stage were all risk factors for poor prognosis. A clinical correlation analysis of the relationship between the five autophagy-related lncRNAs and patients' age, pathological grade, and FIGO stage was also per https://orcid.org/0000-0001-7090-1750 formed. Histopathological assessment of the tumor microenvironment showed that the ESTIMATE, immune, and stromal scores in the high-risk group were lower than those in the low-risk group. Principal component analysis and functional annotation were performed to confirm the correlations. To further evaluate the effect of the model constructed on prognosis, samples were divided into training (60%) and validation (40%) groups, regarding the risk status as an independent prognostic risk factor. A prognostic nomogram was constructed using patients' age, pathological grade, FIGO stage, and risk status to estimate the patients' survival rate. C-index and multi-index ROC curves were generated to verify the stability and accuracy of the nomogram. From this analysis, we concluded that the five lncRNAs identified in this study could affect the incidence and development of endometrial cancer by regulating the autophagy process. Therefore, these molecules may have the potential to serve as novel therapeutic targets and biomarkers.

## 1. Introduction

Endometrial cancer is one of the most common malignant tumors among women. In 2020, this cancer was the fourth most common malignant tumor in American women, and 65,620 new cases and 12,590 deaths annually were predicted [1]. The most common clinical manifestation of endometrial cancer is irregular vaginal bleeding [2], and common risk factors include advanced age, obesity, reproductive issues, and hormone replacement therapy [3–6]. Endometrial cancer is often divided into two types. The estrogen-related Type I has a good prognosis; whereas, Type II is unrelated to estrogen, differentiates poorly, and is more aggressive [7].

Long noncoding RNA (lncRNA) is an RNA that is ≥200 nucleotides [8]. lncRNA participates in the incidence and development of several diseases such as cardiovascular disease, nervous system diseases, and malignant tumors [9–11]. There are several mechanisms of lncRNA regulation in the incidence and development of malignancies [12–14]. For example, in triple-negative breast cancer, let-7b acts as a decoy, allowing HOST2 to repress STAT3 expression, and thus, regulate tumor proliferation and migration [15].

Autophagy is the process in which cells phagocytose and degrade their own components to satisfy the cell's own metabolic needs and to renew certain organelles. Autophagy is also related to the incidence and development of cardiovascular disease, neurodegenerative diseases, and malignant tumors [16–18], although the role of autophagy in malignancies has not been elucidated yet. However, some studies indicate that autophagy can promote the progression of malignant tumors and inhibit their incidence and development [19, 20]. In mammary cancer, for example, autophagy-mediated degradation of NBR1 restricts metastasis [21].

Several advances in the study of autophagy have also been made in endometrial cancer. For example, inhibition of autophagy results in progestin resistance via the PI3K/AKT/mTOR pathway [22]. Several articles about screening immune-related lncRNAs to construct prognostic models through a coexpression method had been published [23, 24]. Likewise, using a coexpression method, we identified five autophagy-related lncRNAs *in silico* by collating and merging data on endometrial cancer samples in The Cancer Genome Atlas (TCGA) database (https://portal.gdc.cancer.gov/) [25] and data on autophagy-related genes in the Human Autophagy Database (HADb) (http://www.autophagy.lu/) [26]. We then constructed a prognostic nomogram to estimate the patients' survival rate and concluded that these lncRNAs have potential as novel therapeutic targets and tumor biomarkers.

## 2. Materials and Methods

### 2.1. Autophagy-Related lncRNAs

We obtained the list of autophagy-related genes from HADb [26]. Fragments per kilobase million (FPKM) RNA-seq data of 575 endometrial cancer samples, which included 23 normal samples and 552 tumor samples, were downloaded from the TCGA database (https://portal.gdc.cancer.gov/) [25]. The Ensembl human genome browser, GRCh38.p13 (http://asia.ensembl.org/index.html), was used to annotate and classify the lncRNAs and protein-coding genes. The expression matrix of all genes was generated by data processing and divided into an lncRNA expression matrix and an mRNA expression matrix. By integrating autophagy-related genes with the mRNA expression matrix, the expression matrix of autophagy-related genes was produced. Autophagy-related lncRNAs and the expression matrix of autophagy-related lncRNAs were analyzed using the limma package in R software. The autophagy-related lncRNAs were selected based on the criteria that the absolute value of the correlation coefficient was greater than 0.6 (|*R*| > 0.6) and the *P* value was less than 0.001 (*P* < 0.001).

### 2.2. Cox Regression and Survival Analysis

Clinical data for endometrial cancer samples were downloaded from UCSC Xena (https://xenabrowser.net/) [25, 27, 28] and organized and merged with the expression matrix of autophagy-related lncRNAs. Using the survival package in R software, univariate and multivariate Cox regression analyses were performed to obtain a list of autophagy-related lncRNAs associated with prognosis. The hazard ratios (HRs) were used to identify risk-related lncRNAs (HR > 1) and protective lncRNAs (HR < 1). Next, the samples were divided into high-risk and low-risk groups based on the risk score: risk score = ∑_*i*=1_^*n*^coef(*i*) × *x*(*i*), where coef(*i*) and *x*(*i*) represent the estimated regression coefficient and the expression value of each autophagy-related lncRNA, respectively. Then, the survival and survminer packages in R software were used to draw survival curves for the two groups. The pheatmap package in R software was used to draw risk curves for the high-risk and low-risk groups.

### 2.3. Independent Prognostic Analysis and Clinical Correlation Analysis

The survival package in R software was used to conduct univariate and multivariate independent prognostic analyses to evaluate the effects of age, pathological grade, and FIGO stage on prognosis. The survivalROC package in R software was used to draw a multi-index ROC curve to assess the accuracy of the constructed model. Then, the ggpubr package in R software was used for clinical correlation analysis.

### 2.4. Tumor Microenvironment Scores and Principal Component Analysis

Based on the ESTIMATE algorithm, the limma and estimate packages in R software were used to calculate ESTIMATE, immune, and stromal scores in the tumor microenvironment for all samples. By collating and merging clinical data, ESTIMATE, immune, and stromal scores for different risk statuses were then obtained. Next, R software was used to draw box plots of immune and stromal scores for different risk statuses. Principal component analysis was then performed on all risk-associated genes and immune-related lncRNAs using limma and scatterplot3d packages in R software.

### 2.5. Functional Annotation

Gene sets REACTOME_AUTOPHAGY (systematic name: M27935), GO_REGULATION_OF_AUTOPHAGY (systematic name: M10281), GO_NEGATIVE_REGULATION_OF_AUTOPHAGY (systematic name: M12149), and GO_MACROAUTOPHAGY (systematic name: M11871) were downloaded from the gene set enrichment analysis (GSEA) database (https://www.gsea-msigdb.org/gsea/index.jsp). Then, the expression matrix of all genes and clinical data for different risk statuses were sorted and merged. Next, gene set enrichment analysis of these four gene sets was performed using GSEA (4.0.2) software [29, 30]. The enriched gene sets were obtained based on a *P* value < 0.05 and a false discovery rate (FDR) value < 0.25 after performing 1,000 permutations.

### 2.6. Multivariate Cox Regression Analysis and Nomogram

All samples were divided into training (60%) and validation (40%) groups using the foreign, survival, and caret packages in R software. Multivariate Cox regression analysis was performed with the rms, foreign, and survival packages in R software. The nomogram was constructed using the rms, foreign, and survival packages in R software. We then used the concordance index (C-index) to evaluate the discrimination and predictive abilities of the nomogram. The range of the C-index value was 0.5 to 1.0. A higher C-index indicates greater discrimination ability of the predicting mode. Then, survival and timeROC packages in R software were used to draw a multi-index ROC curve for the training and validation groups.

### 2.7. Data Statistics

All statistical analyses were performed using R software (R-3.6.1) and strawberry-Perl-5.30.0.1. *P* values < 0.05 were considered statistically significant.

## 3. Results

### 3.1. Univariate and Multivariate Cox Regression Analysis

**Figure 1:**
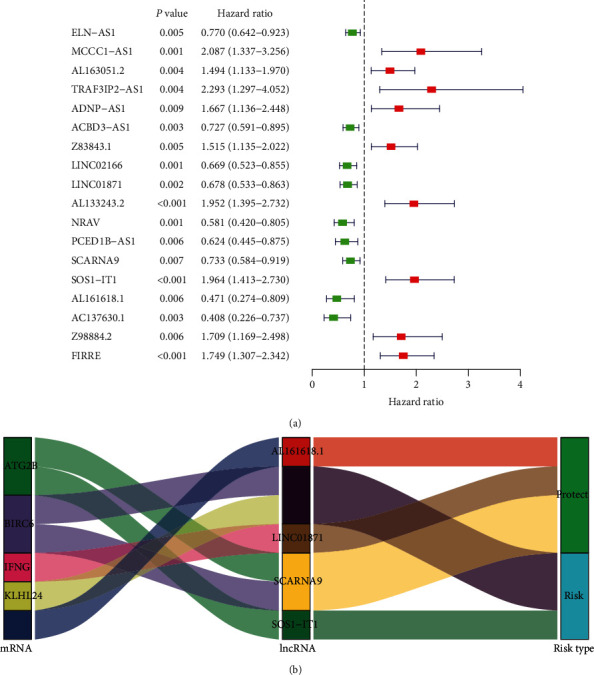
Univariate Cox regression analysis. (a) Forest plot of autophagy-related lncRNAs. The *P* value, hazard ratio, and associated 95% confidence interval for the autophagy-related lncRNAs are shown in the plot. Red indicates a risk-associated lncRNA (HR > 1) and green indicates a protective lncRNA (HR < 1). (b) Sankey coexpression diagram. The left column represents autophagy-related genes, the middle column represents autophagy-related lncRNAs, and the right column represents risk types.

To identify autophagy-related lncRNAs associated with prognosis, transcript data for endometrial cancer samples and autophagy-related genes were integrated. This revealed 32 autophagy-related genes and 171 autophagy-related lncRNAs which were coexpressed. For the autophagy-related lncRNAs, univariate Cox regression analysis was conducted, and a forest map was constructed, revealing 18 autophagy-related lncRNAs associated with prognosis (Figure 1(a)). As shown in Figure 1, AC137630.1, AL161618.1, NRAV, PCED1B-AS1, LINC02166, LINC01871, ACBD3-AS1, SCARNA9, and ELN-AS1 were protective, while AL163051.2, Z83843.1, ADNP-AS1, Z98884.2, FIRRE, AL133243.2, SOS1-IT1, MCCC1-AS1, and TRAF3IP2-AS1 were associated with risk. Multivariate Cox regression analysis identified five autophagy-related lncRNAs (Table 1). The endometrial cancer samples were divided into high-risk and low-risk groups based on the median of risk scores, calculated using the following function: risk score = (expression level of LINC01871×−0.292) + (expression level of SCARNA9×−0.284) + (expression level of SOS1 − IT1 × 0.414) + (expression level of AL161618.1×−0.703) + (expression level of FIRRE × 0.379). The coexpression relationship between the five autophagy-related lncRNAs and autophagy-related genes was visualized on a Sankey diagram (Figure 1(b)).

Regression coefficients, *P* value, hazard ratio, and associated 95% confidence interval for the autophagy-related lncRNAs are shown.

### 3.2. Survival Analysis and Risk Curves

**Figure 2:**
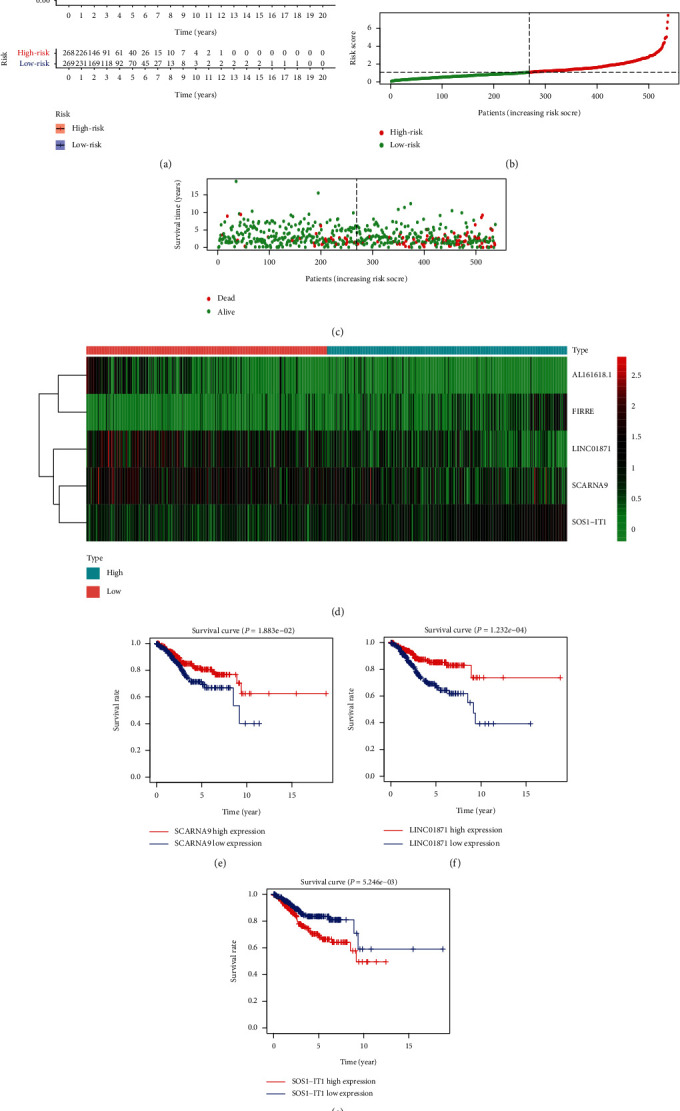
Survival analysis and risk curves. (a) A survival curve for endometrial cancer. Red indicates the high-risk group and blue indicates the low-risk group. (b) A risk curve for endometrial cancer. Red is the high-risk group and green is the low-risk group. (c) A scatter plot of different survival statuses of endometrial cancer patients. Red dots denote patients that died and green dots denote patients that survived. (d) Hierarchical clustering of five autophagy-related lncRNA expression levels. Differences in expression levels of five autophagy-related lncRNAs in different risk statuses. Red indicates the low-risk group and blue indicates the high-risk group. (e–g) Survival curves of autophagy-related lncRNAs. Blue represents the low expression group and red represents the high expression group.

To compare the differences in overall survival rates between different risk statuses, a survival curve was plotted (Figure 2(a)). As shown in Figure 2, the overall survival rate of the high-risk group was significantly lower than the low-risk group. Risk curves for the two groups (Figures 2(b) and 2(c)) showed that the risk value of the high-risk group was higher than that of the low-risk group and the survival time of patients that died was shorter than that of surviving patients. To compare the expression levels of the five autophagy-related lncRNAs in different risk states, a heat map was plotted (Figure 2(d)). As shown in Figure 2, the expression levels of FIRRE and SOS1-IT1 in the high-risk group were higher than those in the low-risk group, while the expression levels of AL161618.1, LINC01871, and SCARNA9 in the high-risk group were lower than those in the low-risk group. Survival curves of these five lncRNAs (Figures 2(e)–2(g)) showed that low survival rates were associated with low expressions of SCARNA9 and LINC01871 and high expressions of SOS1-IT1.

### 3.3. Independent Prognostic Analysis and Clinical Correlation Analysis

**Figure 3:**
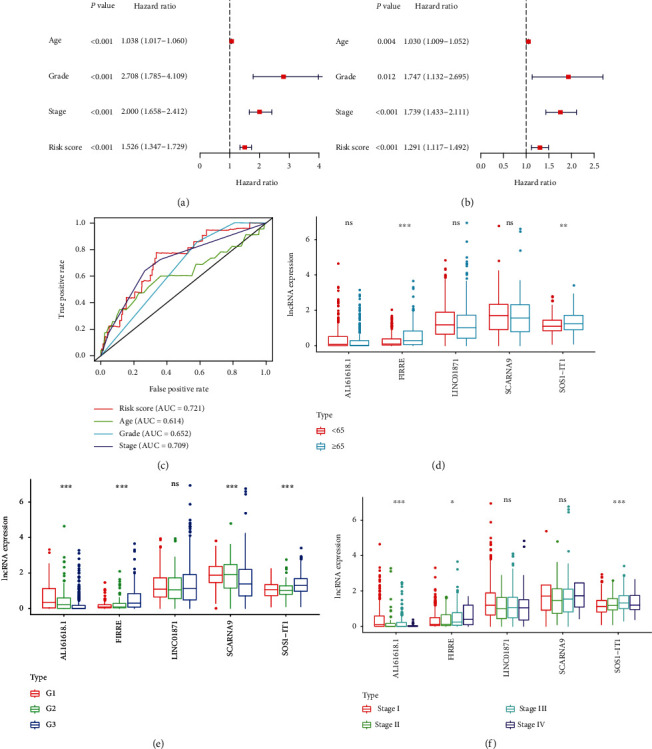
Independent prognostic analysis and clinical correlation analysis. (a) A Forest plot of univariate independent prognostic analysis. The *P* value and hazard ratios and associated 95% confidence intervals are shown in the plot. Red indicates a risk-associated factor (HR > 1) and green indicates a protective factor (HR < 1). (b) A Forest plot of multivariate independent prognostic analysis. The *P* value and hazard ratios and associated 95% confidence intervals are shown in the plot. Red indicates a risk-associated factor (HR > 1) and green indicates a protective factor (HR < 1). (c) Multi-index ROC curve. Risk score AUC = 0.721; age AUC = 0.614; grade AUC = 0.652; stage AUC = 0.709. (d) lncRNA expression level in groups aged below 65 and above 65. Red indicates the group aged under 65 and blue indicates the group aged over 65. (e) lncRNA expression level in different pathologic grades. (f) lncRNA expression level in different FIGO stages. ^∗^*P* < 0.05; ^∗∗^*P* < 0.01; ^∗∗∗^*P* < 0.001. ns, *P* > 0.05.

To analyze the effects of age, pathological grade, FIGO stage, and risk status on prognosis, univariate and multivariate independent prognostic analyses were performed (Figures 3(a) and 3(b)). As shown in Figure 3, patients' age, pathological grade, FIGO stage, and risk status were all risk factors for poor prognosis by univariate and multivariate independent prognostic analyses. A multi-index ROC curve was drawn to evaluate the accuracy of the constructed model (Figure 3(c)). As shown in Figure 3, the risk, age, pathological grade, and FIGO stage scores (AUC) were 0.721, 0.614, 0.652, and 0.709, respectively. Subsequently, a clinical correlation analysis to evaluate the correlation between the five autophagy-related lncRNAs and the patients' age, pathological grade, and FIGO stage was performed (Figures 3(d)–3(f)). This analysis showed that expressions of FIRRE and SOS1-IT1 were associated with the patient's age, pathological grade, and FIGO stage, while AL161618.1 was associated with the patient's pathological grade and FIGO stage. SCARNA9 was associated with the patient's pathological grade.

### 3.4. Tumor Microenvironment Score and Principal Component Analysis

**Figure 4:**
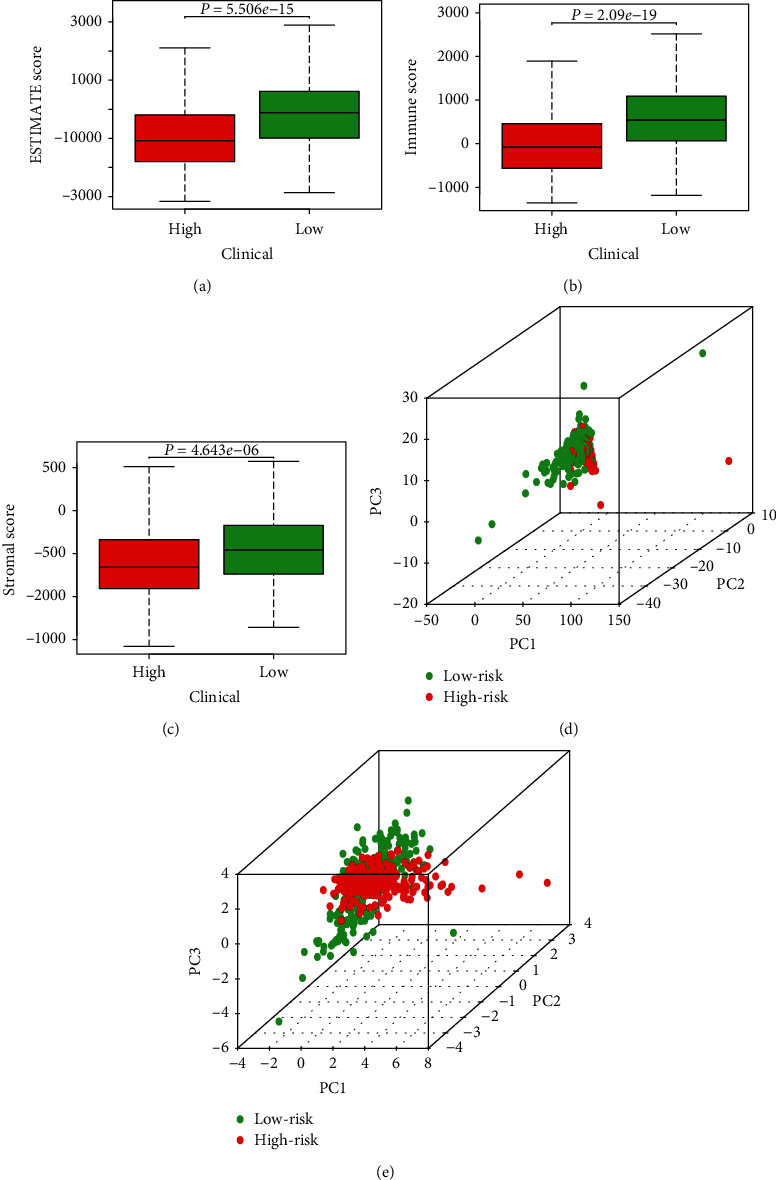
Tumor microenvironment score and principal component analysis. (a) ESTIMATE scores for different risk statuses. The box-plot shows a significant difference between high- and low-risk groups (*P* < 0.05). (b) Immune scores for different risk statuses. The box-plot shows a significant difference between high- and low-risk groups (*P* < 0.05). (c) Stromal scores for different risk statuses. The box-plot shows a significant difference between high- and low-risk groups (*P* < 0.05). (d, e) Principal component analysis of low- and high-risk groups based on autophagy-related lncRNAs and risk-associated genes. Red indicates the high-risk group and green indicates the low-risk group.

The tumor microenvironment is complex and closely related to the incidence and development of tumors [31–34]. Notably, autophagy is also closely related to the tumor microenvironment. For example, by inhibiting autophagy, cadherin-6 facilitates epithelial mesenchymal transition (EMT) and cancer metastasis in thyroid cancer [35]. In this study, we calculated the ESTIMATE, immune, and stromal scores of endometrial cancer samples and merged these scores with collated clinical data and generated box plots of ESTIMATE, immune, and stromal scores for different risk statuses (Figures 4(a)–4(c)). As shown in Figure 4, the medians of ESTIMATE, immune, and stromal scores in the low-risk group were higher than those in the high-risk group. Principal component analysis based on the expression of autophagy-related lncRNAs and risk-associated genes (Figures 4(d) and 4(e)) showed that the separation between the high- and low-risk groups was significant.

### 3.5. Functional Annotation

**Figure 5:**
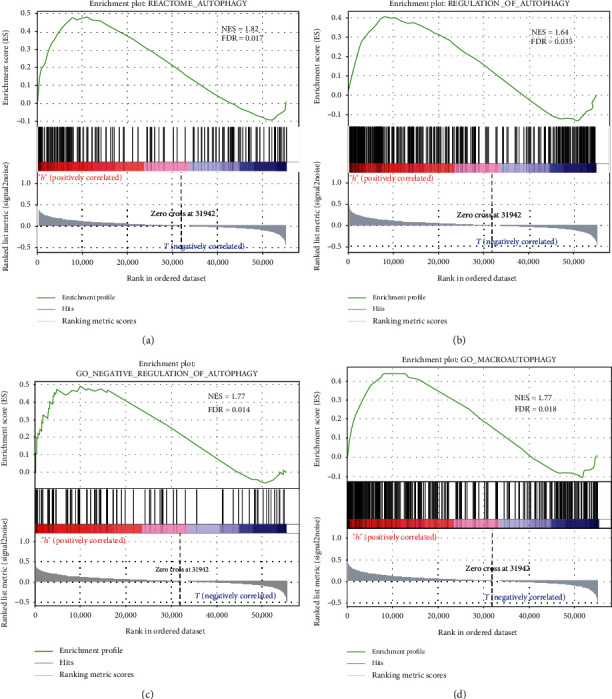
Functional annotation. (a–d) Gene set enrichment analysis (GSEA) indicated significant enrichment of the autophagy-related phenotype in the high-risk patients.

To functionally annotate the five autophagy-related lncRNAs, gene set enrichment analysis of four autophagy-related gene sets was performed (Figures 5(a)–5(d)). As shown in Figure 5, the high-risk group was better enriched in the gene sets than the low-risk group. Therefore, we concluded that the five autophagy-related lncRNAs were associated with the autophagy process.

### 3.6. Multivariate Cox Regression Analysis and Nomogram

**Figure 6:**
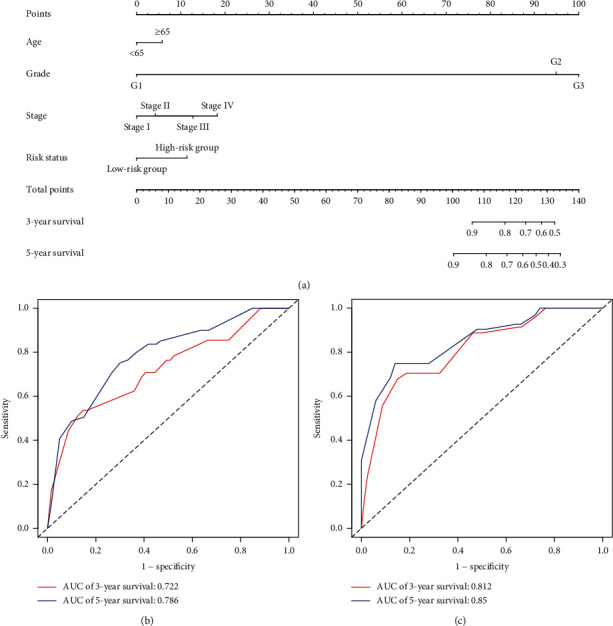
Multivariate Cox regression analysis and nomogram. (a) A survival nomogram. An individual patient's value is located on each variable axis, and a vertical upward line determines the number of points received for each variable value. The sum of these numbers is located on the “Total points” axis, and a vertical downward line determines the likelihood of 3- or 5-year survival. (b) A multi-index ROC curve for training samples. Red indicates 3-year survival and blue indicates 5-year survival. (c) A multi-index ROC curve for validation samples. Red indicates 3-year survival and blue indicates 5-year survival.

To further evaluate the effect of the model constructed based on the five autophagy-related lncRNAs on prognosis, we divided all tumor samples into training (60%) and validation (40%) groups, taking risk status as an independent prognostic risk factor. Multivariate Cox regression analysis to evaluate the correlation between patients' age, pathological grade, FIGO stage, risk status, and prognosis (Table 2) showed that pathological grade, FIGO stage, and risk status were associated with a patient's prognosis. The survival rate of the patient was estimated on a nomogram using patients' age, pathological grade, FIGO stage, and risk status (Figure 6(a)). The accuracy of the nomogram was assessed using the C-index and multi-index ROC curve for the training group (Figure 6(b)). The C-index, 3-year survival, and 5-year survival AUC values were 0.737 (standard error ± 0.037), 0.722, and 0.786, respectively, confirming the accuracy of the nomogram. The stability of the nomogram was evaluated using the C-index and multi-index ROC curve of the verification group (Figure 6(c)). The C-index, 3-year survival, and 5-year survival AUC values were 0.831 (standard error ± 0.032), 0.812, and 0.85, respectively, validating the stability of the nomogram.

Age ≥ 65 was compared to age < 65; Grade 2 and Grade 3 were compared to Grade 1; Stage II, Stage III, and Stage IV were compared to Stage I; the high-risk group was compared to the low-risk group. Regression coefficients, *P* value, hazard ratio, and 95% confidence interval of the clinical characteristics are shown.

## 4. Discussion

Autophagy can regulate the occurrence and development of malignant tumors in various ways. For example, in human breast cancer, autophagy induction is enhanced via cell growth suppression by integral membrane protein 2A [36] PAQR3 inhibits tumor progression in NSCLC cells by modulating EGFR-regulated autophagy [37]. In addition, melatonin/PGC1A/UCP1 promotes tumor slimming and restrains tumor progression by initiating autophagy and lipid browning [38]. It has been established that lncRNA also regulates the development of tumors. For example, in ovarian cancer, lncRNA RHPN1-AS1 acts as a competing endogenous RNA (ceRNA) against miR-596 and upregulates LETM1, promoting tumorigenesis and metastasis [39]. In triple-negative breast cancer, lncRNA HUMT hypomethylation activates FOXK1 transcription, promoting lymphangiogenesis and metastasis [40]. Interestingly, lncRNA can also regulate the occurrence and development of tumors by regulating autophagy. For example, in breast cancer, lncRNA RNA H19 induces autophagy activation via the H19/SAHH/DNMT3B axis, contributing to tamoxifen resistance [41]. FOXP1-induced lncRNA CLRN1-AS1 inactivates the Wnt/*β*-catenin signaling pathway, suppressing autophagy and proliferation in pituitary prolactinoma [42].

In this study, we mined TCGA and HADb for data on endometrial cancer samples and found five autophagy-related lncRNAs (LINC01871, SCARNA9, SOS1-IT1, AL161618.1, and FIRRE). Of these five autophagy-related lncRNAs, the functions of LINC01871, SOS1-IT1, and AL161618.1 are not clear; however, SCARNA9 is downregulated in cervical cancer [43]. FIRRE is associated with spontaneous regression of neuroblastoma [44]. In diffuse large B-cell lymphoma, FIRRE promotes tumor development by activating the Wnt/*β*-catenin signaling pathway [45]. lncRNA biomarkers discovered in endometrial cancer have universal applicability [46, 47]. In contrast, we discovered that lncRNAs could regulate the progress of tumors by regulating autophagy in endometrial cancer, which is important to the study of its mechanism. However, the functions of these lncRNAs in endometrial cancer have not been studied. Using a bioinformatics approach, we found that the five lncRNAs may regulate the occurrence and development of endometrial cancer by regulating autophagy. We also estimated the patients' survival rate using a nomogram, but the specific role and mechanism of these lncRNAs in endometrial cancer remains unknown. We identified five autophagy-related lncRNAs which have potential as new tumor biomarkers and therapeutic targets, but their mechanisms need to be further explored.

## 5. Conclusion

By mining the information on endometrial cancer samples in the TCGA database, we found five autophagy-related lncRNAs and constructed a risk model based on the five autophagy-related lncRNAs. The differences in tumor microenvironment scores in different risk statuses were also compared. Finally, we drew a nomogram to estimate patients' survival rates using the patients' age, pathological grade, FIGO stage, and risk status.

## Figures and Tables

**Table 1:** Multivariate Cox regression analysis of the five autophagy-related lncRNAs.

lncRNA	Coefficient	HR	HR.95L	HR.95H	*P* value
LINC01871	-0.292	0.747	0.590	0.946	0.015
SCARNA9	-0.284	0.753	0.608	0.931	0.009
SOS1-IT1	0.414	1.514	1.060	2.162	0.023
AL161618.1	-0.703	0.495	0.287	0.855	0.012
FIRRE	0.379	1.461	1.037	2.059	0.030

**Table 2:** Multivariate Cox regression analysis of clinical characteristics.

Variable	Coefficient	HR	Lower.95	Upper.95	*P* value
Age ≥ 65	0.464	1.590	0.896	2.822	0.113
Grade 2	2.070	7.927	1.009	62.253	0.049
Grade 3	2.074	7.954	1.070	59.142	0.043
Stage II	0.611	1.843	0.771	4.404	0.169
Stage III	0.818	2.265	1.173	4.374	0.015
Stage IV	1.406	4.080	1.749	9.517	0.001
High-risk group	0.866	2.377	1.255	4.502	0.008

## Data Availability

We obtained RNA-seq data from TCGA (http://cancergenome.nih.gov/) and clinical information from UCSC Xena (https://xenabrowser.net). All gene sets were downloaded from GSEA (http://software.broadinstitute.org/gsea/index.jsp).

## Abbreviations

TCGA: The Cancer Genome Atlas
HADb: Human Autophagy Database
GSEA: Gene set enrichment analysis
HR: Hazard ratio
RNA-seq: RNA sequencing
NES: Normalized enrichment score.
